# Impaired pulmonary function mediates the impact of preterm birth on later-life stroke: a 2-step, multivariable Mendelian randomization study

**DOI:** 10.4178/epih.e2023031

**Published:** 2023-03-03

**Authors:** Xingzhi Guo, Peng Tang, Chen Hou, Yue Liu, Rui Li

**Affiliations:** 1 Department of Geriatric Neurology, Shaanxi Provincial People’s Hospital, Xi’an, China; 2 Shaanxi Provincial Clinical Research Center for Geriatric Medicine, Xi’an, China; 3 Institute of Medical Research, Northwestern Polytechnical University, Xi’an, China

**Keywords:** Stroke, Preterm birth, Forced expiratory volume in the first second, Gestational age, Mendelian randomization

## Abstract

**OBJECTIVES:**

Observational studies have suggested an association between preterm birth and stroke in late adulthood, but it remains unclear whether the association is causal. The purpose of this study was to evaluate the causal effects of gestational age on stroke and to determine the pathophysiological mechanisms underlying the causal associations.

**METHODS:**

Two-sample Mendelian randomization (MR) was performed to assess the causal effects of fetal gestational duration, early preterm birth (EPB), preterm birth, or postterm birth on stroke and its subtypes. Two-step Mendelian randomization (TSMR) and multivariable Mendelian randomization (MVMR) were additionally used to determine the role of common stroke risk factors, including cardiovascular diseases, hypertension, pulmonary impairment, inflammation, and metabolic diseases, in mediating the causal associations between gestational age and stroke and its subtypes.

**RESULTS:**

Genetically predicted EPB increased the risk of cardioembolic stroke (CES; odds ratio [OR], 1.115; 95% confidence interval [CI], 1.036 to 1.200; p=0.004) and large artery stroke (LAS; OR, 1.131; 95% CI, 1.031 to 1.241; p=0.009). The TSMR results showed that EPB was associated with a lower forced expiratory volume in the first second and forced vital capacity ratio (FEV1/FVC) (β=-0.020; 95% CI, -0.035 to -0.005; p=0.009), which increased the risk of CES and LAS. Further MVMR analysis showed that the associations between EPB and stroke disappeared after adjustment for FEV1/FVC.

**CONCLUSIONS:**

Our data demonstrate that EPB is causally associated with an elevated risk of CES and LAS, and that pulmonary dysfunction mediates the causal impact of EPB on CES and LAS.

## INTRODUCTION

Stroke is one of the leading causes of disability and death globally [1]. Thus, identifying the potential risk factors for stroke is essential for reducing its social and economic burden worldwide [2]. Epidemiological studies have shown that approximately 15 million preterm births (PBs) occur every year worldwide [3]. Multiple observational studies have suggested that PB may be associated with stroke risk in adulthood, but the results were inconsistent [4-6]. The results of observational studies are often distorted by confounding factors (e.g., the genetic and environmental background) and reverse causation [7]. Thus, it is crucial to determine the causal relationship between PB and the risk for later-life stroke. Moreover, the potential mediator(s) contributing to the elevated risk of later-life stroke in individuals born preterm need to be identified.

Previous studies have indicated that preterm delivery was associated with an increased risk of cardiovascular diseases (e.g., abnormal blood pressure and atrial fibrillation), pulmonary dysfunction, systematic inflammation, lipid metabolism dysfunction, obesity, and type 2 diabetes mellitus in children and adults [8-12]. most of which are established risk factors for stroke. For example, the lung is an organ that matures in the later stage of pregnancy, making preterm children susceptible to pulmonary dysfunction and complications [13]. The forced expiratory volume in the first second (FEV1), forced vital capacity (FVC), and their ratio (FEV1/FVC) are the 3 main indices reflecting pulmonary function. Clinical data have shown that PB children had significantly lower values of FEV1 and FVC [14], which were strongly associated with susceptibility to stroke. However, little is known about the key mediator(s) between PB and future susceptibility to stroke in adulthood.

Mendelian randomization (MR) analysis, which treats genetic variants as instrumental variables (IVs), can be used to assess the causal relationship between exposures and the corresponding outcome. To address the above issues, we conducted a 2-sample MR analysis to evaluate the causal associations between genetically predicted gestational age and stroke in later life. Moreover, both 2-step Mendelian randomization (TSMR) and multivariable Mendelian randomization (MVMR), which could explain whether an exposure-outcome association is mediated by other factors were performed to determine the pathophysiological mechanisms underlying the causal associations [15].

## MATERIALS AND METHODS

### Study design and data source

Genome-wide association studies (GWAS) presenting summarylevel data of European ancestry for gestational age were obtained from the Early Growth Genetics (EGG) Consortium [16], including full gestational duration (GD; n= 84,689), early preterm birth (EPB; < 34 weeks, n = 1,139), PB ( < 37 weeks, n = 4,775), and postterm birth (PoB; ≥ 42 weeks, n= 7,888) using spontaneous delivery (≥ 39 and < 42 weeks) as the reference [16]. Summary statistics for stroke were obtained from the MEGASTROKE Consortium and International Stroke Genetics Consortium [17,18], including overall stroke (n= 446,696), large artery stroke (LAS; n= 150,765), cardioembolic stroke (CES; n= 211,763), any ischemic stroke (AIS; n= 440,328), small vessel stroke (SVS; n= 198,048), and intracerebral hemorrhage (ICH; n= 6,948). For potential mediators linking gestation age and stroke, GWAS datasets for pulmonary function (FEV1 and FEV1/FVC ratio) were obtained from the Within Family Consortium (https://www.withinfamilyconsortium.com/) using datasets of related individuals, data on highdensity lipoprotein (HDL) cholesterol and low-density lipoprotein (LDL) cholesterol were obtained from United Kingdom Biobank [19], data on total cholesterol were obtained from Global Lipids Genetics Consortium [20], data on body mass index (BMI) and obesity classification (class 1, ≥ 30 kg/m^2^; class 2, ≥ 35 kg/m^2^; class 3, ≥ 40 kg/m^2^) were obtained from the Genetic Investigation of ANthropometric Traits [21,22], and data on systolic blood pressure (SBP) and diastolic blood pressure (DBP) were obtained from the International Consortium of Blood Pressure [23]. Summary statistics of atrial fibrillation (AF), type 2 diabetes (T2D), and C-reactive protein (CRP) were obtained from the GWAS meta-analysis performed by Ligthart et al. [24], Nielsen et al. [25], and Xue et al. [26]. The detailed characteristics of each GWAS dataset included in this study, including those for mediators, are summarized in Supplementary Material 1.

### Mendelian randomization analysis

In order to obtain a sufficient number of IVs for gestational age, single-nucleotide polymorphisms (SNPs) with a p-value less than 1E–06 were picked as IVs for all traits set as exposures. The IVs were clumped according to the 1000 Genomes Project linkage disequilibrium structure (r^2^< 0.01, within 1,000 kb, European). Only SNPs with an F-statistic greater than 10 were maintained for the MR analysis, since this threshold is considered to be indicative of strong instruments and reduces the risk of bias due to a weak instrument bias [27,28]. A detailed list of all harmonized IVs with F-statistic values for each exposure-outcome group is presented in the Supplementary Material 2. Causal effects were evaluated using the TwoSampleMR version 0.5.6 in R using the inverse variance weighted (IVW) method [27]. The Mendelian Randomization version 0.6.0 was also applied to calculate the estimates using the Egger and Lasso methods in MVMR [29]. Statistical significance was defined as a p-value lower than 0.05.

**Figure 1.**
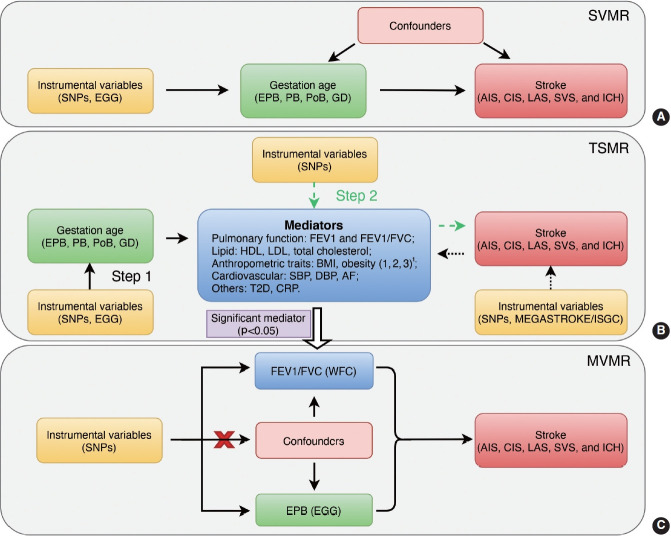
Flowchart of the MR analysis conducted in this study. (A) The MR analysis investigated the effect of gestational age on stroke. (B) TSMR analysis evaluated pulmonary function as a potential mediator between gestational age and stroke. Bidirectional MR analysis assessed the causal effects of stroke on mediators (dashed arrow, B). (C) MVMR analysis investigated the role of pulmonary function in mediating the effect of EPB on stroke. MR, Mendelian randomization; SVMR, single-variable Mendelian randomization; TSMR, 2-step Mendelian randomization; MVMR, multivariable Mendelian randomization; SNPs, single-nucleotide polymorphisms; FEV1, forced expiratory volume in the first second; FEV1/FVC, forced expiratory volume in the first second/forced vital capacity; HDL, high density lipoprotein; LDL, low-density lipoprotein; EPB, early preterm birth; PB, preterm birth; PoB, postterm birth; GD, gestational duration; AIS, any ischemic stroke; LAS, large artery stroke; CES, cardioembolic stroke; SVS, small vessel stroke; ICH, intracerebral hemorrhage; ISGC, International Stroke Genetics Consortium; EGG, early growth genetics; WFC, Within Family Consortium; CRP, C-reactive protein; BMI, body mass index; SBP, systolic blood pressure; DBP, diastolic blood pressure; T2D, type 2 diabetes; AF, atrial fibrillation. 1Obesity classes 1, 2, and 3 are defined as BMI ≥30 kg/m^2^, ≥35 kg/m^2^, and ≥40 kg/m^2^ individually.

For TSMR, we first (step 1) evaluated the causal effects of gestational age (EPB, PB, PoB, and GD) on potential mediators, and then (step 2) assessed the causal effects of potential mediators on stroke. Bidirectional MR was also performed to estimate the reverse causal effects of stroke and its subtypes on mediators. For MVMR, we combined the IVs of the gestational age and positive mediator(s) identified in the TSMR analysis, namely EPB and FEV1/FVC in this study, to further determine to what extent FEV1/FVC mediated the causal effect of EPB on stroke (Figure 1).

### Heterogeneity, power, and sensitivity analysis

The Cochran Q-statistic was applied to evaluate heterogeneity in the MR analysis. To validate the strength and reliability of the MR results, the F-statistic was calculated. The MR-Pleiotropy RESidual Sum and Outlier (MR-PRESSO) test and MR-Egger intercept were applied to identify horizontal pleiotropic outliers [30,31]. Leave-one-out analysis was conducted to estimate the impact of a single SNP on the overall causal effect by removing 1 SNP each time.

### Ethics statement

No patients were directly involved in the overall process of our study. This study was performed based on publicly available data and no separate ethical approval was required.

## RESULTS

### Causal estimates between gestational age and stroke

**Figure 2.**
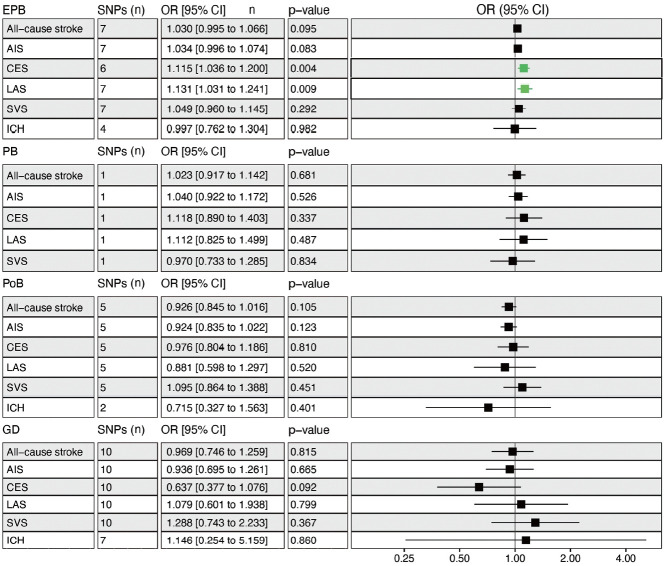
Mendelian randomization analysis to determine the causal effects of gestational age on stroke. Effect sizes with 95% CIs, numbers of SNPs, and p-values are presented. Genetically predicted EPB increased the risk of CES and LAS. No causal effects of PB, PoB, or GD on stroke were found. SNPs, single-nucleotide polymorphisms; OR, odds ratio; CI, confidence interval; EPB, early preterm birth; PB, preterm birth; PoB, postterm birth; GD, gestational duration; AIS, any ischemic stroke; LAS, large artery stroke; CES, cardioembolic stroke; SVS, small vessel stroke; ICH, intracerebral hemorrhage.

Genetically determined EPB was significantly associated with an increased risk of CES (odds ratio [OR], 1.115; 95% confidence interval [CI], 1.036 to 1.200; p= 0.004) and LAS (OR, 1.131; 95% CI, 1.031 to 1.241; p= 0.009), but not overall stroke (Figure 2). Leave-one-out permutation analysis showed that rs112912841 (in the mapped *LPP* gene), which was strongly associated with EPB [16], drove the main effect in the causal estimate for LAS (Supplementary Material 3), but no evidence of pleiotropy and heterogeneity was found in the MR-PRESSO global test and Cochran Q-test (Supplementary Material 4). No significant causal relationship was observed between PB, PoB, GD, and stroke, and there was no obvious heterogeneity (Figure 2, Supplementary Material 4). Leaveone-out permutation analysis showed the same trend after removing each palpable sensitive SNP (Supplementary Materials 5 and 6).

### Causal estimates between gestational age and mediators

**Figure 3.**
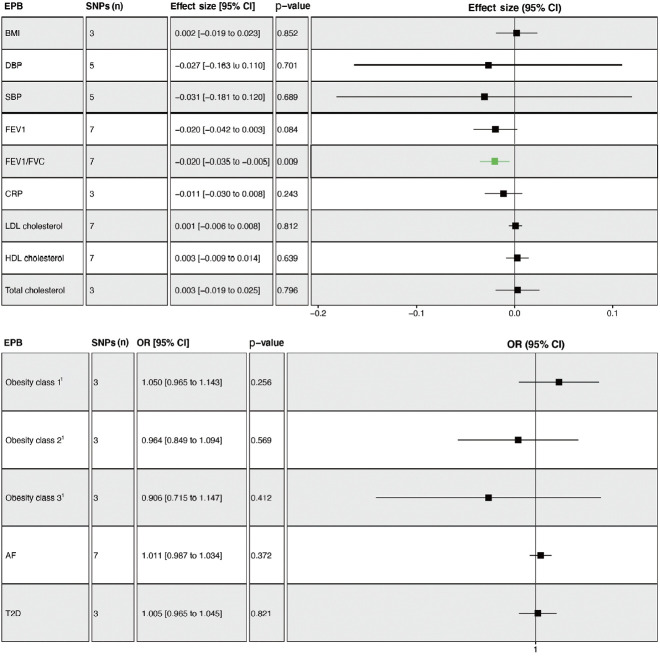
Mendelian randomization analysis to determine the causal effects of EPB on mediators. Effect sizes with 95% CIs, numbers of SNPs, and p-values are presented. Genetically predicted EPB decreased the FEV1/FVC ratio in adulthood, but not other mediators. EPB, early preterm birth; SNPs, single-nucleotide polymorphisms; FEV1, forced expiratory volume in the first second; FEV1/FVC, forced expiratory volume in the first second/forced vital capacity; HDL, high density lipoprotein; LDL, low-density lipoprotein; CRP, C-reactive protein; BMI, body mass index; SBP, systolic blood pressure; DBP, diastolic blood pressure; T2D, type 2 diabetes; AF, atrial fibrillation; OR, odds ratio; CI, confidence interval. ^1^Obesity classes 1, 2, and 3 are defined as BMI ≥30 kg/m^2^, ≥35 kg/m^2^, and ≥40 kg/m^2^ individually.

The overall results showed that EPB was only associated with a lower FEV1/FVC ratio (β= -0.020; 95% CI, -0.035 to -0.005; p= 0.009) (Figure 3). Leave-one-out permutation analysis showed that rs112912841, which was strongly associated with EPB [16], drove the main effect in the causal estimate for FEV1/FVC, without obvious pleiotropy and heterogeneity (Supplementary Materials 7 and 8). Similarly, no significant causal effects of PB and GD on any of the potential mediators were observed (Supplementary Materials 9 and 10). PoB was associated with higher BMI (β= 0.048; 95% CI, 0.011 to 0.085; p= 0.011) and lower HDL cholesterol levels (β= -0.038; 95% CI, -0.066 to -0.010; p= 0.008) (Supplementary Material 11).

### Reciprocal estimates between mediators and stroke

**Figure 4.**
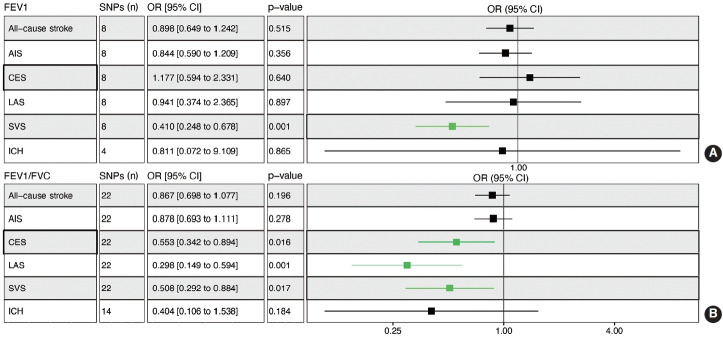
Mendelian randomization analysis to determine the causal effects of pulmonary function on stroke. Effect sizes with 95% CIs, numbers of SNPs, and p-values are presented. (A) Genetically predicted high FEV1 decreased the risk of SVS in adulthood. (B) Genetically predicted high FEV1/FVC ratio decreased the risk of CES, LAS, and SVS in adulthood. OR, odds ratio; CI, confidence interval; SNPs, single-nucleotide polymorphisms; FEV1, forced expiratory volume in the first second; FEV1/FVC, forced expiratory volume in the first second/forced vital capacity; AIS, any ischemic stroke; LAS, large artery stroke; CES, cardioembolic stroke; SVS, small vessel stroke; ICH, intracerebral hemorrhage.

Genetically determined FEV1 was associated with a reduced risk of SVS (OR, 0.410; 95% CI, 0.248 to 0.678; p= 0.001) (Figure 4A). Leave-one-out permutation analysis showed that no single SNP drove the causal estimates for FEV1, with obvious heterogeneity (Supplementary Materials 12 and 13). The FEV1/FVC ratio was associated with a lower risk of CES (OR, 0.553; 95% CI, 0.342 to 0.894; p = 0.016), LAS (OR, 0.298; 95% CI, 0.149 to 0.594; p= 0.001), and SVS (OR, 0.508; 95% CI, 0.292 to 0.884; p= 0.017) but not overall stroke, AIS, or ICH (Figure 4B, Supplementary Materials 13 and 14). BMI, SBP, DBP, total cholesterol, obesity, AF, and T2D were associated with a higher risk of stroke, while HDL cholesterol was associated with a lower risk of stroke (Supplementary Material 15).

Genetically determined SVS was associated with a decreased risk of FEV1 (β= -0.036; 95% CI, -0.070 to -0.002; p= 0.037), with horizontal pleiotropy in MR-Egger regression (Supplementary Material 16). No significant causal effects of stroke and its subtypes on the FEV1/FVC ratio were found (Supplementary Materials 16-19). Genetically determined stroke and its subtypes were associated with SBP, DBP, AF, and TD (Supplementary Materials 20 and 21).

### The causal effect of early preterm birth and forced expiratory volume in the first second/forced vital capacity on stroke in multivariable Mendelian randomization

The results of MVMR showed that FEV1/FVC remained significantly associated with a reduced risk of CES (OR_IVW_, 0.606; 95% CI, 0.394 to 0.931; p= 0.022) and LAS (OR_IVW_, 0.314; 95% CI, 0.161 to 0.612; p< 0.001). However, the causal effect of EPB on LAS disappeared (OR_IVW_, 1.088; 95% CI, 0.984 to 1.204; p= 0.324), but it remained for CES (OR_IVW_, 1.110; 95% CI, 1.040 to 1.184; p= 0.001), suggesting that FEV1/FVC entirely and partially mediated the estimates of EPB on LAS and CES, respectively. Consistent results were obtained using the MR-Egger and Lasso approach (Table 1).

## DISCUSSION

Increasing evidence has suggested that women with an abnormal-term pregnancy (< 37 or ≥ 42 weeks) have an increased risk of future stroke, but the impact of gestational age on stroke in adult offspring is still largely undetermined. Through an MR analysis, we found that infants with a history of EPB had a higher risk of LAS and CES than those with spontaneous delivery. In addition, our results obtained using the TSMR and MVMR methods suggested that pulmonary dysfunction (low FEV1/FVC) might mediate the effect of EPB on LAS and CES in adults born preterm.

A recent cosibling study with over 2 million singletons showed that PB, especially EPB, was associated with a higher risk of stroke in adulthood [6]. Similarly, our results showed that only PB less than 34 weeks (namely, EPB), but not 37 weeks (PB), was causally linked to an elevated risk of LAS and CES. Interestingly, Ueda et al. [5] found that infants born before 32 weeks, but not between 32 weeks and 36 weeks, had a nearly 2-fold increased risk of cerebrovascular disease (hazard ratio, 1.89) compared to term-born individuals. Although the impact of EPB on stroke was only significant in LAS and CES, we found that EPB infants also had an elevated risk of overall stroke (p= 0.095) and AIS (p= 0.083) that almost reached statistical significance, suggesting that EPB may have a broad effect on stroke later in life. Indeed, previous studies have shown that people born at an early gestational age were prone to developing cerebrovascular disease and specifically occlusive stroke [32]. It is unclear whether gestational age between 34 weeks (EPB) and 37 weeks (PB) plays a role in regulating the development and maturation of the circulatory system in the brain of the fetus [33].

Epidemiological studies have found that preterm delivery was associated with multiple risk factors for stroke, such as cardiovascular disease, lipid metabolism disorders, chronic inflammation, and pulmonary dysfunction [8-10,12]. However, in this study, we found that EPB was only associated with a decreased FEV1/FVC ratio, causally leading to an elevated risk of stroke. These data indicate that EPB children with a lower FEV1/FVC ratio probably have an elevated risk of stroke in adulthood. A potential reason contributing to this phenomenon may be that the lung is one of the last organs to mature during pregnancy, and preterm neonates often suffer from a variety of respiratory symptoms owing to lung immaturity and pulmonary atelectasis caused by the lack of pulmonary surfactant. For example, bronchopulmonary dysplasia is a chronic lung disease in premature infants, causing an increased risk of adverse respiratory outcomes throughout life [34]. A similar study with a larger sample size demonstrated that preterm children had decreasing FEV1 and FEV1/FVC scores in a time-dependent manner, manifested by at least a 0.1 z-score decline per year in children [35].

Our MVMR results suggested that the causal effect of EPB on LAS and CES in late adulthood was entirely and partially mediated by the FEV1/FVC ratio, respectively. Chronic obstructive pulmonary disease (COPD) is characterized by chronic airflow limitation and respiratory symptoms and is diagnosed based on a low FEV1/FVC ratio [36]. Multiple studies have revealed that compared to reference individuals, patients with COPD had a higher risk of overall stroke as well as all stroke subtypes, including ischemic stroke, intracerebral hemorrhage, and subarachnoid hemorrhage [37]. A recent prospective cohort study showed that, compared to term delivery, PB was significantly associated with a lower FEV1/FVC ratio and a higher risk of COPD at the age of 53 years (OR, 2.9) [36]. These data remind clinicians that improving pulmonary function in EPB infants could reduce stroke risk later in life.

The results of this study should be interpreted cautiously owing to the limitations described below. First, to ensure that there were adequate IVs for gestational age, we set the p-value threshold for IV selection to 1E-06 in this MR study, which may have introduced weak instrument bias to the overall estimates. Second, only 1 SNP was available as an IV for PB, which might weaken the causal effects of PB on stroke in MR analysis. Third, the results of this study were based on GWAS summary statistics from people of European descent, and whether the identified relationship is a common phenomenon in other ethnic populations should be further evaluated. Fourth, the case sample size of the EBP GWAS was small, and further studies with larger sample sizes are warranted.

In conclusion, our data demonstrate that genetically predicted EPB is causally associated with an increased risk of future CES and LAS in adulthood. In addition, the results of TSMR and MVMR suggest that pulmonary dysfunction mediates the causal effects of EPB on the risk of CES and LAS in adulthood. Our findings suggest that positively promoting pulmonary maturity and improving pulmonary function might be beneficial ways to prevent future stroke in individuals with an EPB history.

## DATA AVAILABILITY

In this study, the GWAS summary statistics for GD are available from the EGG Consortium (https://egg-consortium.org/). GWAS summary statistics for stroke and its subtypes are available from the MEGASTROKE consortium (http://megastroke.org/) and OpenGWAS (https://gwas.mrcieu.ac.uk/). The GWAS datasets for FEV1, FEV1/FVC ratio, HDL cholesterol, LDL cholesterol, total cholesterol, BMI, SBP, DBP, AF, T2D, CRP, and obesity classification (class 1: ≥30 kg/m^2^; class 2: ≥35 kg/m^2^; class 3: ≥40 kg/m^2^) are available from OpenGWAS (https://gwas.mrcieu.ac.uk/). The R scripts used in this study are available from the authors upon request.

## Figures and Tables

**Table 1.** MVMR results of EPB and the FEV1/FVC ratio on the risk of stroke

Exposures	Method	All-cause stroke			AIS			CES			LAS			SVS			ICH		
		SNPs (n)	OR (95% CI)	p-value	SNPs (n)	OR (95% CI)	p-value	SNPs (n)	OR (95% CI)	p-value	SNPs (n)	OR (95% CI)	p-value	SNPs (n)	OR (95% CI)	p-value	SNPs (n)	OR (95% CI)	p-value
EPB	Egger	26	1.052 (0.998, 1.109)	0.055	26	1.063 (1.005, 1.126)	0.033	25	1.156 (1.033, 1.292)	0.011	26	1.145 (0.979, 1.340)	0.089	26	1.095 (0.956, 1.253)	0.191	18	1.012 (0.705, 1.451)	0.950
	IVW	26	1.021 (0.995, 1.046)	0.101	26	1.026 (0.995, 1.058)	0.100	26	1.110 (1.040, 1.184)	0.001	26	1.088 (0.984, 1.204)	0.324	26	1.006 (0.942, 1.074)	0.848	18	0.985 (0.787, 1.232)	0.897
	Lasso	26	1.021 (0.985, 1.057)	0.235	26	1.026 (0.988, 1.065)	0.181	25	1.109 (1.029, 1.195)	0.006	26	1.088 (0.983, 1.205)	0.100	26	1.006 (0.921, 1.098)	0.887	18	0.985 (0.781, 1.241)	0.900
FEV1/FVC	Egger	26	0.862 (0.687, 1.082)	0.203	26	0.890 (0.695, 1.139)	0.360	25	0.581 (0.351, 0.959)	0.034	26	0.299 (0.151, 0.591)	0.001	26	0.485 (0.272, 0.865)	0.014	18	0.468 (0.118, 1.856)	0.280
	IVW	26	0.893 (0.759, 1.049)	0.171	26	0.928 (0.761, 1.131)	0.461	26	0.606 (0.394, 0.931)	0.022	26	0.314 (0.161, 0.612)	6.62E-04	26	0.526 (0.344, 0.803)	0.002	18	0.482 (0.131, 1.771)	0.272
	Lasso	26	0.893 (0.714, 1.116)	0.321	26	0.928 (0.728, 1.184)	0.549	25	0.606 (0.370, 0.993)	0.047	26	0.314 (0.161, 0.612)	0.001	26	0.526 (0.297, 0.930)	0.027	18	0.482 (0.126, 1.836)	0.285

MVMR, multivariable mendelian randomization; EPB, early preterm birth; FEV1/FVC, forced expiratory volume in the first second/forced vital capacity; AIS, any ischemic stroke; CES, cardioembolic stroke; LAS, large artery stroke; SVS, small vessel stroke; ICH, intracerebral hemorrhage; IVW, inverse variance-weighted; SNP, single-nucleotide polymorphisms; OR, odds ratio; CI, confidence interval.

## SUPPLEMENTARY MATERIALS

Supplementary Material 1. Summary of the GWAS datasets included in this MR study.

Supplementary Material 2 Summary of harmonized intrumental variables with F-statistic values for each exposure-outcome group.

Supplementary Material 3 Forest plots of leave-one out sensitivity analysis of EPB on stroke.

Supplementary Material 4 Heterogeneity and pleiotropy analysis of gestational duration on the risk of stroke

Supplementary Material 5 Forest plots of leave-one out sensitivity analysis of PoB on stroke.

Supplementary Material 6 Forest plots of leave-one out sensitivity analysis of GD on stroke.

Supplementary Material 7 Forest plots of leave-one out sensitivity analysis of EPB on pulmonary function.

Supplementary Material 8 Heterogeneity and pleiotropy analysis of gestational duration on pulmonary function

Supplementary Material 9 MR analysis determine the causal effects of PB on mediators.

Supplementary Material 10 MR analysis determine the causal effects of GD on mediators.

Supplementary Material 11 MR analysis determine the causal effects of PoB on mediators.

Supplementary Material 12 Forest plots of leave-one out sensitivity analysis of FEV1 on stroke.

Supplementary Material 13 Heterogeneity and pleiotropy analysis of pulmonary function on the risk of stroke

Supplementary Material 14 Forest plots of leave-one out sensitivity analysis of FEV1/FVC on stroke.

Supplementary Material 15 MR analysis determine the causal effects of potential mediators on stroke.

Supplementary Material 16 Heterogeneity and pleiotropy analysis of stroke on pulmonary function

Supplementary Material 17 MR analysis determine the causal effects of stroke on pulmonary function.

Supplementary Material 18 Forest plots of leave-one out sensitivity analysis of stroke on FEV1.

Supplementary Material 19 Forest plots of leave-one out sensitivity analysis of stroke on FEV1/FVC.

Supplementary Material 20 MR analysis determine the causal effects of stroke on continuous mediators.

Supplementary Material 21 MR analysis determine the causal effects of stroke on binary mediators.
