# Supplementary material for: Impaired pulmonary function mediates the impact of preterm birth on later-life stroke: a 2-step, multivariable Mendelian randomization study
Source: Epidemiol Health. 2023 Mar 3;45:e2023031. doi: 10.4178/epih.e2023031 (PMC10586927; doi:10.4178/epih.e2023031)
Supplement: Supplementary Material 3 — Forest plots of leave-one out sensitivity analysis of EPB on stroke. [file epih-45-e2023031-Supplementary-3.docx]

**
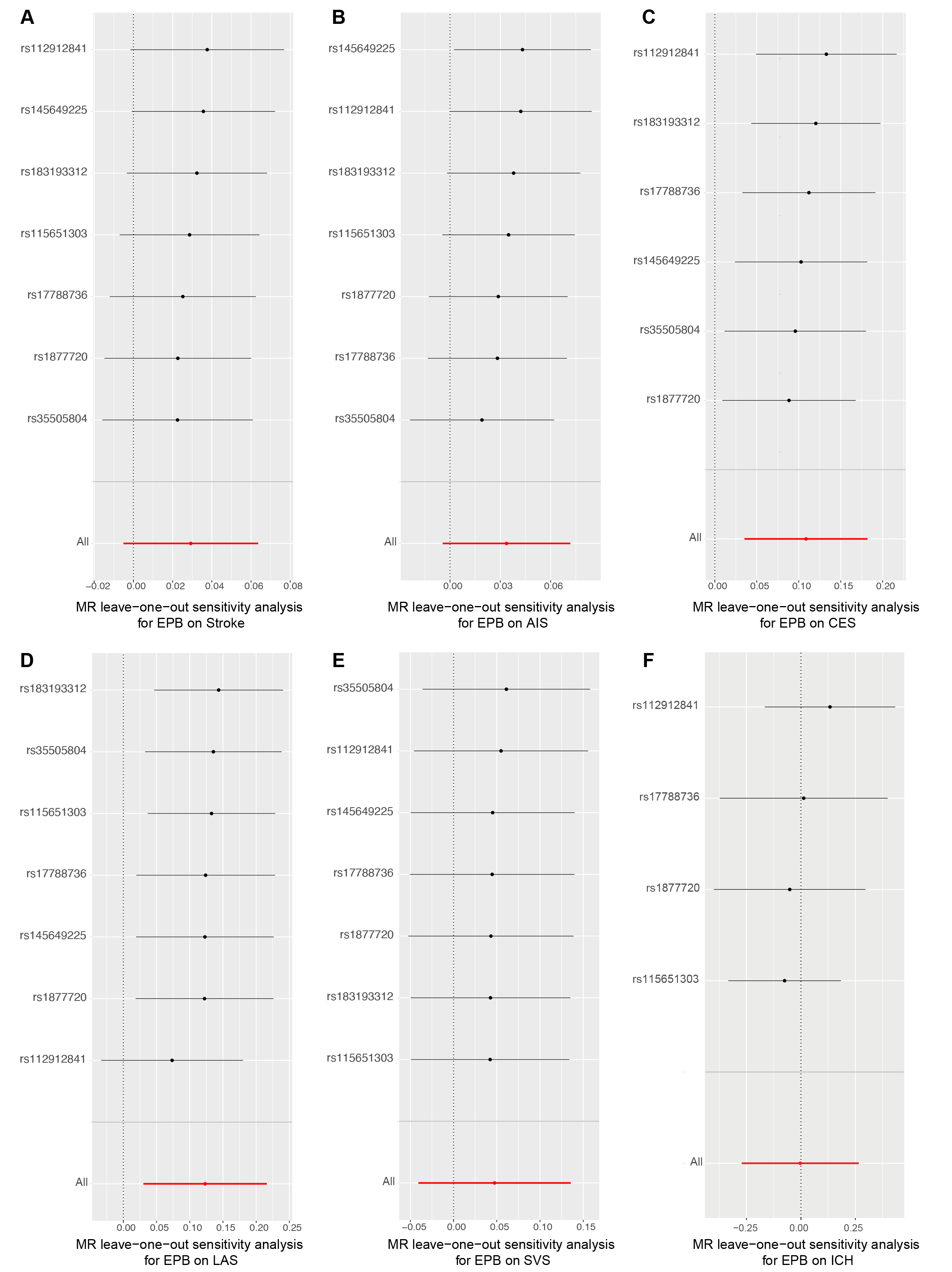
**

**Supplementary Material 3. Forest plots of leave-one out sensitivity analysis of EPB on stroke**. Using IVW method, leave-one-out analysis showed the causal effect of EPB on all cause stroke (**A**), AIS (**B**), CES (**C**), LAS (**D**), SVS (**E**), and ICH (**F**) individually. Each SNP was iteratively excluded in MR analysis. EPB, early preterm birth; LAS, large artery stroke; CES, cardioembolic stroke; SVS, small vessel stroke; AIS, any ischemic stroke; ICH, intracerebral hemorrhage; MR, Mendelian randomization.
