# Supplementary material for: Impaired pulmonary function mediates the impact of preterm birth on later-life stroke: a 2-step, multivariable Mendelian randomization study
Source: Epidemiol Health. 2023 Mar 3;45:e2023031. doi: 10.4178/epih.e2023031 (PMC10586927; doi:10.4178/epih.e2023031)
Supplement: Supplementary Material 4 — Heterogeneity and pleiotropy analysis of gestational duration on the risk of stroke [file epih-45-e2023031-Supplementary-4.docx]

Supplementary Material 4. Heterogeneity and pleiotropy analysis of gestational duration on the risk of stroke

| **Outcome** | **Method** |  | **Any stroke^*^** | | |  | **AIS^*^** | | |  | **CES^*^** | | |  | **LAS^*^** | | |  | **SVS^*^** | | |  | **ICH^#^** | | |
| --- | --- | --- | --- | --- | --- | --- | --- | --- | --- | --- | --- | --- | --- | --- | --- | --- | --- | --- | --- | --- | --- | --- | --- | --- | --- |
|  |  |  | MR-Egger  intercept (P) | Cochran-Q  (P) | MR_PRESSO (P) |  | MR-Egger intercept (P) | Cochran-Q (P) | MR_PRESSO (P) |  | MR-Egger intercept (P) | Cochran-Q (P) | MR_PRESSO (P) |  | MR-Egger intercept (P) | Cochran-Q (P) | MR_PRESSO (P) |  | MR-Egger intercept (P) | Cochran-Q (P) | MR_PRESSO (P) |  | MR-Egger intercept (P) | Cochran-Q (P) | MR_PRESSO (P) |
| **EPB** | MR-Egger |  | -0.02(0.584) | 2.89(0.715) | 4.58(0.772) |  | -0.04(0.315) | 3.64(0.601) | 7.06(0.554) |  | -0.01(0.834) | 3.74(0.441) | 5.53(0.623) |  | 0.15(0.187) | 3.02(0.695) | 7.84(0.506) |  | 0.03(0.766) | 0.66(0.984) | 1.05(0.992) |  | -0.66(0.316) | 2.00(0.367) | 6.74(0.385) |
|  | IVW |  |  | 3.23(0.778) |  |  |  | 4.88(0.558) |  |  |  | 3.79(0.579) |  |  |  | 5.35(0.499) |  |  |  | 0.76(0.993) |  |  |  | 3.75(0.288) |  |
|  |  |  |  |  |  |  |  |  |  |  |  |  |  |  |  |  |  |  |  |  |  |  |  |  |  |
| **PB** | Wald ratio |  | NA | NA | NA |  | NA | NA | NA |  | NA | NA | NA |  | NA | NA | NA |  | NA | NA | NA |  | NA | NA | NA |
|  |  |  |  |  |  |  |  |  |  |  |  |  |  |  |  |  |  |  |  |  |  |  |  |  |  |
| **PoB** | MR-Egger |  | -0.003(0.855) | 3.09(0.377) | 4.96(0.538) |  | -0.004(0.825) | 3.58(0.309) | 5.77(0.509) |  | 0.008(0.814) | 2.99(0.392) | 4.60(0.585) |  | -0.01(0.846) | 9.41(0.024) | 15.46(0.077) |  | 0.03(0.520) | 1.60(0.658) | 3.39(0.716) |  | NA |  | NA |
|  | IVW |  |  | 3.13(0.535) |  |  |  | 3.65(0.454) |  |  |  | 3.05(0.548) |  |  |  | 9.558(0.048) |  |  |  | 2.13(0.711) |  |  |  | 0.34(0.557) |  |
|  |  |  |  |  |  |  |  |  |  |  |  |  |  |  |  |  |  |  |  |  |  |  |  |  |  |
| **GD** | MR-Egger |  | -0.001(0.935) | 13.11(0.107) | 15.67(0.171) |  | 0.004(0.799) | 14.12(0.078) | 17.01(0.135) |  | 0.008(0.769) | 11.89(0.156) | 14.45(0.241) |  | 0.04(0.160) | 5.88(0.660) | 10.24(0.500) |  | -0.009(0.743) | 7.03(0.532) | 8.59(0.640) |  | 0.09(0.626) | 5.78(0.327) | 7.90(0.450) |
|  | IVW |  |  | 13.12(0.156) |  |  |  | 14.24(0.113) |  |  |  | 12.03(0.211) |  |  |  | 8.27(0.506) |  |  |  | 7.15(0.621) |  |  |  | 6.09(0.412) |  |

Note: MR, Mendelian randomization; EPB, early preterm birth; PB, preterm birth; PoB, post-term birth; GD, gestational duration; LAS, large artery stroke; CES, cardioembolic stroke; SVS, small vessel stroke; AIS, any ischemic stroke; ICH, intracerebral hemorrhage; SNPs, single nucleotide polymorphisms; IVW, inverse-variance weighted; MR_PRESSO, Mendelian Randomization Pleiotropy RESidual Sum and Outlier; P, P value; NA, not applicable.

*: GWAS datasets from MEGASTROKE.

#: GWAS dataset from International Stroke Genetics Consortium (ISGC).
