# Supplementary material for: Impaired pulmonary function mediates the impact of preterm birth on later-life stroke: a 2-step, multivariable Mendelian randomization study
Source: Epidemiol Health. 2023 Mar 3;45:e2023031. doi: 10.4178/epih.e2023031 (PMC10586927; doi:10.4178/epih.e2023031)
Supplement: Supplementary Material 5 — Forest plots of leave-one out sensitivity analysis of PoB on stroke. [file epih-45-e2023031-Supplementary-5.docx]

**
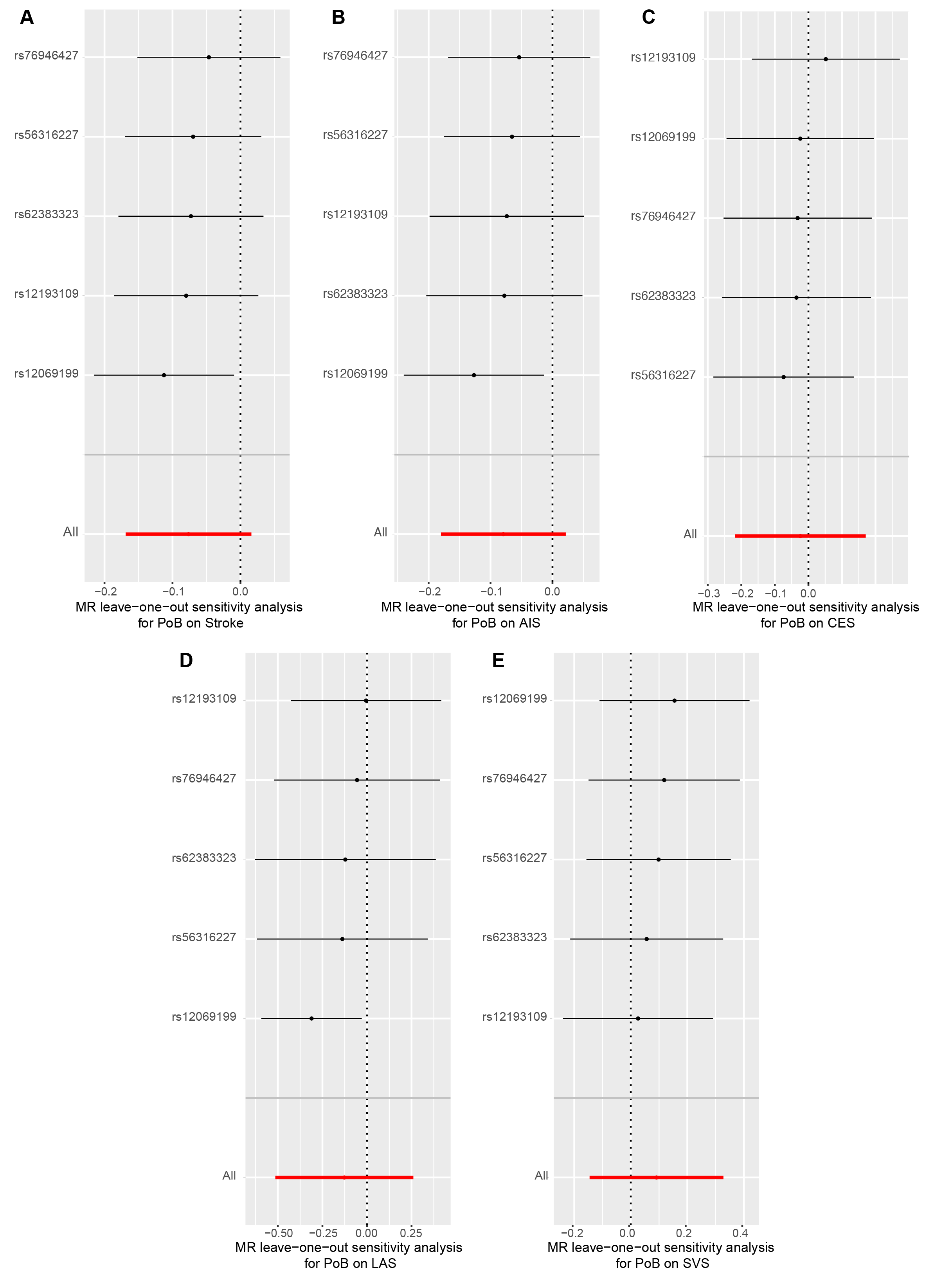
**

**Supplementary Material 5. Forest plots of leave-one out sensitivity analysis of PoB on stroke**. Using IVW method, leave-one-out analysis showed the causal effect of PoB on all cause stroke (**A**), AIS (**B**), CES (**C**), LAS (**D**), and SVS (**E**) individually. Each SNP was iteratively excluded in analysis. PoB, post-term birth; LAS, large artery stroke; CES, cardioembolic stroke; SVS, small vessel stroke; AIS, any ischemic stroke; MR, Mendelian randomization.
