# Supplementary material for: Impaired pulmonary function mediates the impact of preterm birth on later-life stroke: a 2-step, multivariable Mendelian randomization study
Source: Epidemiol Health. 2023 Mar 3;45:e2023031. doi: 10.4178/epih.e2023031 (PMC10586927; doi:10.4178/epih.e2023031)
Supplement: Supplementary Material 7 — Forest plots of leave-one out sensitivity analysis of EPB on pulmonary function. [file epih-45-e2023031-Supplementary-7.docx]

**
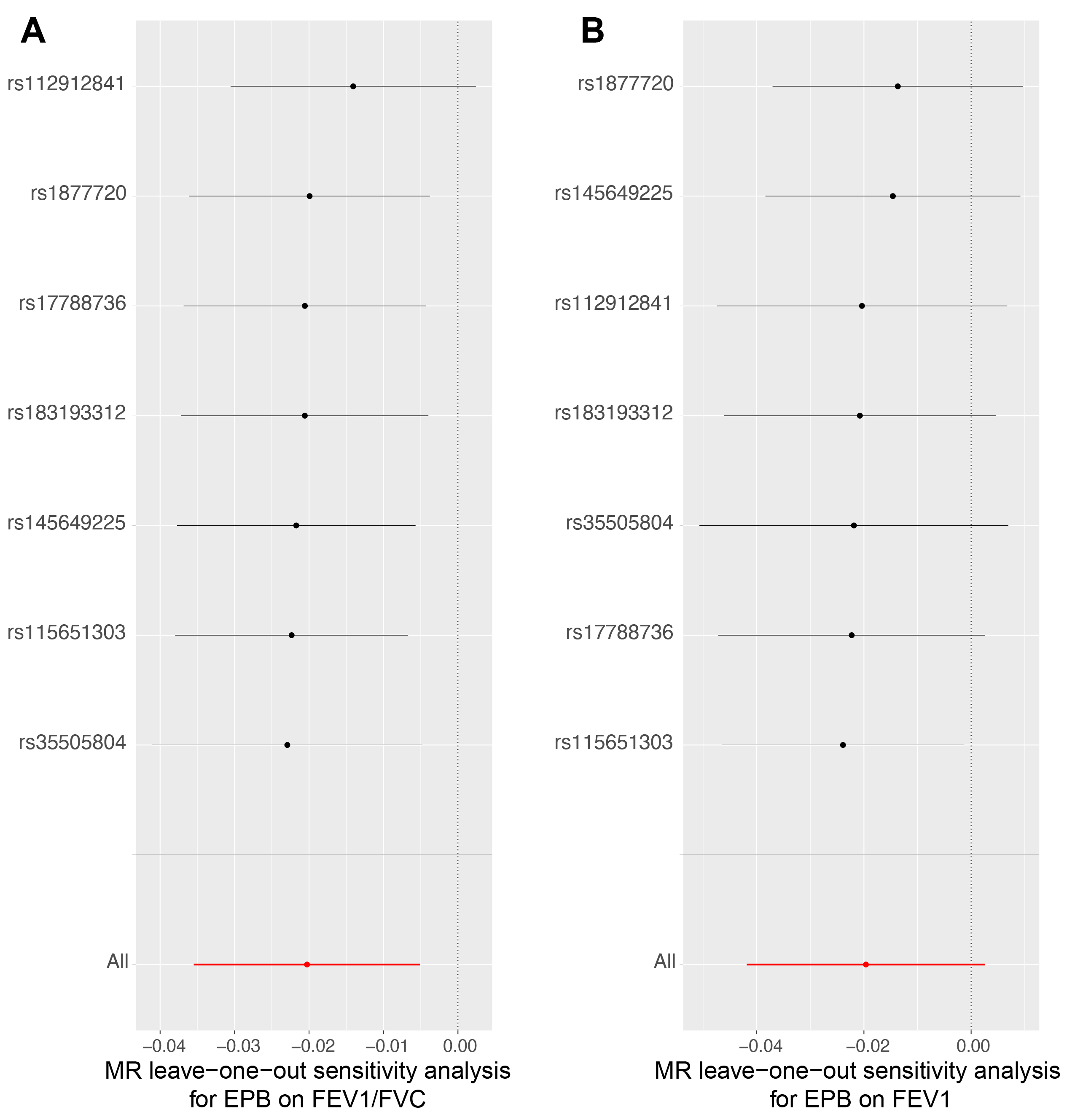
**

**Supplementary Material 7. Forest plots of leave-one out sensitivity analysis of EPB on pulmonary function**. Using IVW method, **A** showed the causal effect between EPB and FEV1/FVC. **B** showed the causal effect between EPB and FEV1. Each SNP was iteratively excluded in analysis. EPB, early preterm birth; FEV1, forced expiratory volume in the first second; FEV1/FVC, forced expiratory volume in the first second/forced vital capacity; MR, Mendelian randomization.
