# Supplementary material for: Impaired pulmonary function mediates the impact of preterm birth on later-life stroke: a 2-step, multivariable Mendelian randomization study
Source: Epidemiol Health. 2023 Mar 3;45:e2023031. doi: 10.4178/epih.e2023031 (PMC10586927; doi:10.4178/epih.e2023031)
Supplement: Supplementary Material 8 — Heterogeneity and pleiotropy analysis of gestational duration on pulmonary function [file epih-45-e2023031-Supplementary-8.docx]

Supplementary Material 8. Heterogeneity and pleiotropy analysis of gestational duration on pulmonary function

| **Exposure\Outcome** | **Method** |  | **FEV1** | | |  | **FEV1/FVC** | | |
| --- | --- | --- | --- | --- | --- | --- | --- | --- | --- |
|  |  |  | MR-Egger intercept (P) | Cochran-Q (P) | MR_PRESSO (P) |  | MR-Egger intercept (P) | Cochran-Q (P) | MR_PRESSO (P) |
| **EPB** | MR-Egger |  | -0.01(0.408) | 5.25(0.385) | 7.49(0.495) |  | -0.01(0.330) | 3.64(0.601) | 6.40(0.629) |
|  | IVW |  |  | 6.10(0.411) |  |  |  | 4.80(0.568) |  |
|  |  |  |  |  |  |  |  |  |  |
| **PB** | Wald ratio |  | NA | NA | NA |  | NA | NA | NA |
|  |  |  |  |  |  |  |  |  |  |
| **PoB** | MR-Egger |  | 0.01(0.377) | 5.91(0.115) | 13.15(0.133) |  | -0.01(0.588) | 8.39(0.038) | 13.62(0.093) |
|  | IVW |  |  | 8.02(0.090) |  |  |  | 9.41(0.051) |  |
|  |  |  |  |  |  |  |  |  |  |
| **GD** | MR-Egger |  | 0.01(0.218) | 16.07(0.041) | 23.91(0.027) |  | 0.009(0.156) | 4.07(0.850) | 9.09(0.599) |
|  | IVW |  |  | 19.65(0.020) |  |  |  | 6.52(0.686) |  |

Note: MR, Mendelian randomization; EPB, early preterm birth; PB, preterm birth; PoB, post-term birth; GD, gestational duration; SNPs, single nucleotide polymorphisms; FEV1, forced expiratory volume in the first second; FEV1/FVC, forced expiratory volume in the first second/forced vital capacity; IVW, inverse-variance weighted; MR_PRESSO, Mendelian Randomization Pleiotropy RESidual Sum and Outlier; P, P value; NA, not applicable.
