# Supplementary material for: Impaired pulmonary function mediates the impact of preterm birth on later-life stroke: a 2-step, multivariable Mendelian randomization study
Source: Epidemiol Health. 2023 Mar 3;45:e2023031. doi: 10.4178/epih.e2023031 (PMC10586927; doi:10.4178/epih.e2023031)
Supplement: Supplementary Material 9 — MR analysis determine the causal effects of PB on mediators. [file epih-45-e2023031-Supplementary-9.docx]

**
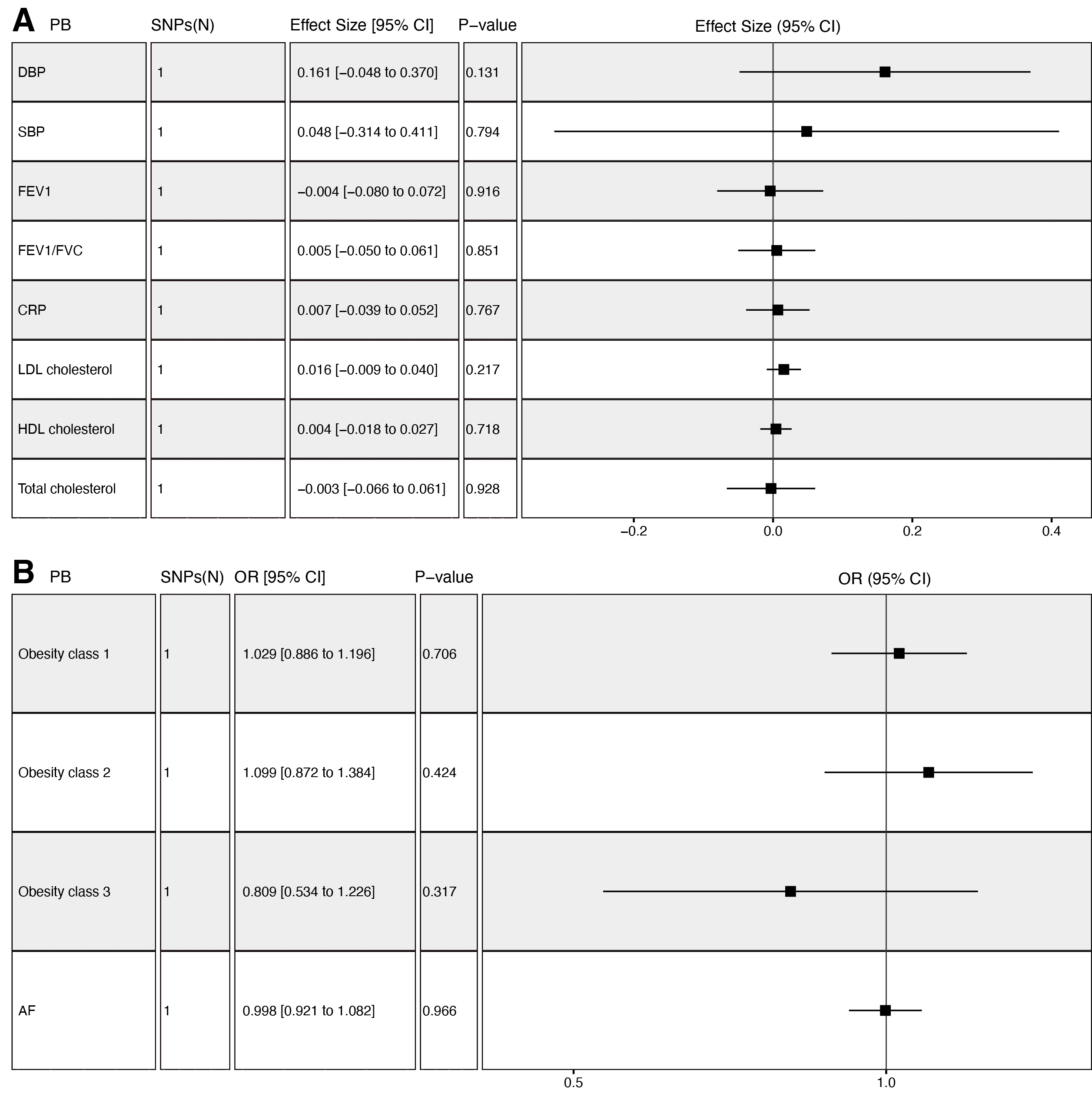
**

**Supplementary Material 9. MR analysis determine the causal effects of PB on mediators.** Causal effect with 95% confidence intervals, number of SNP and P value were presented. **A** showed the effect sizes of genetically predicted PB on continuous outcomes in offspring. **B** showed the causal estimates of genetically predicted PB on binary outcomes in offspring. MR, Mendelian randomization; SNPs, Single nucleotide polymorphisms; FEV1, forced expiratory volume in the first second; FEV1/FVC, forced expiratory volume in the first second/forced vital capacity; PB, preterm birth; CRP, C-reactive protein; SBP, systolic blood pressure; DBP, diastolic blood pressure; AF, atrial fibrillation.
