# Supplementary material for: Impaired pulmonary function mediates the impact of preterm birth on later-life stroke: a 2-step, multivariable Mendelian randomization study
Source: Epidemiol Health. 2023 Mar 3;45:e2023031. doi: 10.4178/epih.e2023031 (PMC10586927; doi:10.4178/epih.e2023031)
Supplement: Supplementary Material 13 — Heterogeneity and pleiotropy analysis of pulmonary function on the risk of stroke [file epih-45-e2023031-Supplementary-13.docx]

Supplementary Material 13. Heterogeneity and pleiotropy analysis of pulmonary function on the risk of stroke

| **Exposure**  **/Outcome** | **Method** |  | **Any stroke^*^** | | |  | **AIS^*^** | | |  | **CES^*^** | | |  | **LAS^*^** | | |  | **SVS^*^** | | |  | **ICH^#^** | | |
| --- | --- | --- | --- | --- | --- | --- | --- | --- | --- | --- | --- | --- | --- | --- | --- | --- | --- | --- | --- | --- | --- | --- | --- | --- | --- |
|  |  |  | MR-Egger  intercept (P) | Cochran-Q  (P) | MR_PRESSO (P) |  | MR-Egger intercept (P) | Cochran-Q (P) | MR_PRESSO (P) |  | MR-Egger intercept (P) | Cochran-Q (P) | MR_PRESSO (P) |  | MR-Egger intercept (P) | Cochran-Q (P) | MR_PRESSO (P) |  | MR-Egger intercept (P) | Cochran-Q (P) | MR_PRESSO (P) |  | MR-Egger intercept (P) | Cochran-Q (P) | MR_PRESSO (P) |
| **FEV1** | MR-Egger |  | -0.03(0.287) | 13.88(0.030) | 23.26(0.025) |  | -0.03(0.422) | 16.12(0.013) | 24.65(0.021) |  | 0.02(0.758) | 17.60(0.007) | 23.67(0.023) |  | -0.16(0.062) | 10.27(0.113) | 24.51(0.016) |  | -0.07(0.171) | 3.24(0.777) | 7.61(0.617) |  | -0.36(0.133) | 2.07(0.353) | 14.99(0.085) |
|  | IVW |  |  | 17.04(0.017) |  |  |  | 18.11(0.011) |  |  |  | 17.90(0.012) |  |  |  | 19.16(0.007) |  |  |  | 5.65(0.580) |  |  |  | 8.33(0.039) |  |
| **FEV1/FVC** | MR-Egger |  | -0.04(0.173) | 8.71(0.985) | 11.67(0.965) |  | -0.04(0.158) | 11.17(0.941) | 14.43(0.887) |  | -0.04(0.477) | 14.43(0.807) | 16.51(0.811) |  | -0.05(0.530) | 27.31(0.126) | 30.59(0.163) |  | 0.006(0.927) | 12.18(0.909) | 13.40(0.936) |  | -0.21(0.457) | 10.17(0.600) | 12.30(0.647) |
|  | IVW |  |  | 10.71(0.968) |  |  |  | 13.32(0.896) |  |  |  | 14.95(0.825) |  |  |  | 27.86(0.143) |  |  |  | 12.19(0.934) |  |  |  | 10.76(0.630) |  |

Note: MR, Mendelian randomization; EPB, early preterm birth; PB, preterm birth; PoB, post-term birth; GD, gestational duration; LAS, large artery stroke; CES, cardioembolic stroke; SVS, small vessel stroke; AIS, any ischemic stroke; ICH, intracerebral hemorrhage; FEV1, forced expiratory volume in the first second; FEV1/FVC, forced expiratory volume in the first second/forced vital capacity; SNPs, single nucleotide polymorphisms; IVW, inverse-variance weighted; MR_PRESSO, Mendelian Randomization Pleiotropy RESidual Sum and Outlier; P, P value; NA, not applicable.

*: GWAS datasets from MEGASTROKE.

#: GWAS dataset from International Stroke Genetics Consortium (ISGC).
