# Supplementary material for: Impaired pulmonary function mediates the impact of preterm birth on later-life stroke: a 2-step, multivariable Mendelian randomization study
Source: Epidemiol Health. 2023 Mar 3;45:e2023031. doi: 10.4178/epih.e2023031 (PMC10586927; doi:10.4178/epih.e2023031)
Supplement: Supplementary Material 16 — Heterogeneity and pleiotropy analysis of stroke on pulmonary function [file epih-45-e2023031-Supplementary-16.docx]

Supplementary Material 16. Heterogeneity and pleiotropy analysis of stroke on pulmonary function

| **Exposure\Outcome** | **Method** |  | **FEV1** | | |  | **FEV1/FVC** | | |
| --- | --- | --- | --- | --- | --- | --- | --- | --- | --- |
|  |  |  | MR-Egger intercept (P) | Cochran-Q (P) | MR_PRESSO (P) |  | MR-Egger intercept (P) | Cochran-Q (P) | MR_PRESSO (P) |
| **Any stroke*** | MR-Egger |  | 0.0008(0.928) | 37.66(0.027) | 40.75(0.039) |  | 0.005(0.382) | 17.82(0.766) | 20.67(0.760) |
|  | IVW |  |  | 37.67(0.037) |  |  |  | 18.61(0.772) |  |
| **AIS*** | MR-Egger |  | 0.002(0.781) | 32.59(0.088) | 35.45(0.137) |  | 0.004(0.799) | 21.90(0.526) | 26.81(0.433) |
|  | IVW |  |  | 32.70(0.110) |  |  |  | 24.19(0.450) |  |
| **CES*** | MR-Egger |  | -0.004(0.572) | 17.42(0.065) | 20.22(0.116) |  | 0.003(0.497) | 5.76(0.834) | 7.44(0.850) |
|  | IVW |  |  | 18.01(0.081) |  |  |  | 6.26(0.855) |  |
| **LAS*** | MR-Egger |  | -0.0006(0.947) | 3.62(0.889) | 4.36(0.952) |  | -0.004(0.563) | 5.98(0.648) | 7.38(0.750) |
|  | IVW |  |  | 3.62(0.934) |  |  |  | 6.34(0.704) |  |
| **SVS*** | MR-Egger |  | -0.019(0.030) | 2.80(0.946) | 12.85(0.358) |  | -0.008(0.236) | 10.88(0.208) | 15.52(0.193) |
|  | IVW |  |  | 9.73(0.372) |  |  |  | 13.10(0.157) |  |
| **ICH^#^** | MR-Egger |  | NA | NA | NA |  | NA | NA | NA |

Note: MR, Mendelian randomization; EPB, early preterm birth; PB, preterm birth; PoB, post-term birth; GD, gestational duration; SNPs, single nucleotide polymorphisms; FEV1, forced expiratory volume in the first second; FEV1/FVC, forced expiratory volume in the first second/forced vital capacity; IVW, inverse-variance weighted; MR_PRESSO, Mendelian Randomization Pleiotropy RESidual Sum and Outlier; P, P value; NA, not applicable.

*: GWAS datasets from MEGASTROKE.

#: GWAS dataset from International Stroke Genetics Consortium (ISGC).
