# Supplementary material for: Impaired pulmonary function mediates the impact of preterm birth on later-life stroke: a 2-step, multivariable Mendelian randomization study
Source: Epidemiol Health. 2023 Mar 3;45:e2023031. doi: 10.4178/epih.e2023031 (PMC10586927; doi:10.4178/epih.e2023031)
Supplement: Supplementary Material 21 — MR analysis determine the causal effects of stroke on binary mediators. [file epih-45-e2023031-Supplementary-21.docx]

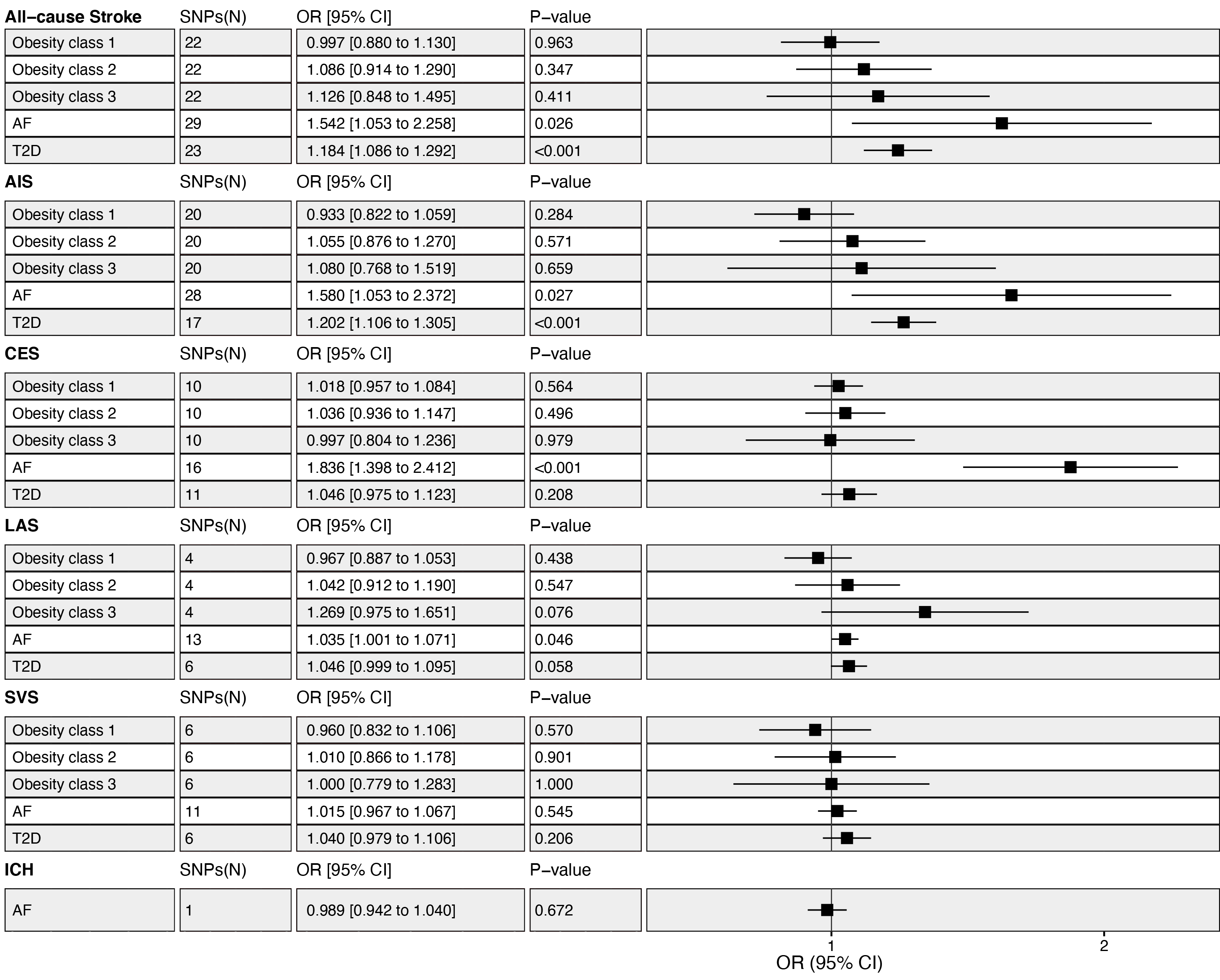


**Supplementary Material 21. MR analysis determine the causal effects of stroke on binary mediators.** Effect sizes with 95% confidence intervals, number of SNP and P value were presented. MR, Mendelian randomization; SNPs, Single nucleotide polymorphisms; LAS, large artery stroke; CES, cardioembolic stroke; SVS, small vessel stroke; AIS, any ischemic stroke; ICH, intracerebral hemorrhage; T2D, type 2 diabetes; AF, atrial fibrillation.
